# Datura genome reveals duplications of psychoactive alkaloid biosynthetic genes and high mutation rate following tissue culture

**DOI:** 10.1186/s12864-021-07489-2

**Published:** 2021-03-22

**Authors:** Alex Rajewski, Derreck Carter-House, Jason Stajich, Amy Litt

**Affiliations:** 1 Department of Botany and Plant Science, University of California, Riverside, California 92521 USA; 2 Department of Microbiology and Plant Pathology, University of California, Riverside, California 92521 USA

**Keywords:** Genome sequencing, *Datura stramonium*, Alkaloids, Tissue culture, Transposable elements, Transformation, Scopolamine

## Abstract

**Background:**

*Datura stramonium* (Jimsonweed) is a medicinally and pharmaceutically important plant in the nightshade family (Solanaceae) known for its production of various toxic, hallucinogenic, and therapeutic tropane alkaloids. Recently, we published a tissue-culture based transformation protocol for *D. stramonium* that enables more thorough functional genomics studies of this plant. However, the tissue culture process can lead to undesirable phenotypic and genomic consequences independent of the transgene used. Here, we have assembled and annotated a draft genome of *D. stramonium* with a focus on tropane alkaloid biosynthetic genes. We then use mRNA sequencing and genome resequencing of transformants to characterize changes following tissue culture.

**Results:**

Our draft assembly conforms to the expected 2 gigabasepair haploid genome size of this plant and achieved a BUSCO score of 94.7% complete, single-copy genes. The repetitive content of the genome is 61%, with *Gypsy*-type retrotransposons accounting for half of this. Our gene annotation estimates the number of protein-coding genes at 52,149 and shows evidence of duplications in two key alkaloid biosynthetic genes, tropinone reductase I and hyoscyamine 6 β-hydroxylase. Following tissue culture, we detected only 186 differentially expressed genes, but were unable to correlate these changes in expression with either polymorphisms from resequencing or positional effects of transposons.

**Conclusions:**

We have assembled, annotated, and characterized the first draft genome for this important model plant species. Using this resource, we show duplications of genes leading to the synthesis of the medicinally important alkaloid, scopolamine. Our results also demonstrate that following tissue culture, mutation rates of transformed plants are quite high (1.16 × 10^− 3^ mutations per site), but do not have a drastic impact on gene expression.

**Supplementary Information:**

The online version contains supplementary material available at 10.1186/s12864-021-07489-2.

## Background

*Datura stramonium* (Jimsonweed) is an important medicinal plant in the nightshade family (Solanaceae) and is known for its production of various tropane alkaloids. These alkaloids primarily consist of hyoscyamine and scopolamine, which are extremely potent anticholinergics that produce hallucinations and delirium; however, they can also be used clinically to counteract motion sickness, irritable bowel syndrome, eye inflammation, and several other conditions [1]. *D. stramonium* is also used extensively in Native American cultures and in Ayurvedic medicine to treat myriad conditions including asthma, ulcers, rheumatism, and many others [2]. While total synthesis of scopolamine and related precursor alkaloids is possible, extraction from plants is currently the most feasible production method [3, 4]. There has been significant interest in genetic engineering or breeding for increased alkaloid content in *D. stramonium*, but like many species, we lack the genetic or genomic tools to enable this [5, 6].

Like many plants, stable genetic engineering of *D. stramonium* requires a complex process of tissue culture, in which phytohormones are used to de-differentiate tissue to form a totipotent mass of cells called a callus. Callus is then transformed and screened for the presence of the transgene using a selectable marker, often an antibiotic resistance gene. Transformed callus is then regenerated into whole plants using phytohormones to induce shoot and later root growth.

Unfortunately, in addition to being very time consuming, this process can have several unwanted genotypic and phenotypic outcomes [7]. Many early studies documented aberrant phenotypes of plants emerging from tissue culture [8, 9]. In the case of tissue culture with transformation, these aberrant phenotypes can be a result of the inserted transgene itself. T-DNA from *Agrobacterium* preferentially integrates into transcriptionally active regions of the genome, and constructs used for transgenic transformation also often contain one or more strong enhancer and promoter elements which can alter transcriptional levels of genes or generate antisense transcripts [10–17]. Insertion of T-DNA sequences has also been shown to disrupt genome structure both on small and large scales, causing deletions, duplications, translocations, and transversion [18–20]. Apart from the direct effects of the transgene insertion, tissue culture is an extremely physiologically stressful process for plant tissue. These exposures to exogenous and highly concentrated phytohormones, antibiotics, and modified (formerly) pathogenic *Agrobacterium* have each been independently documented to cause changes in development and to alter the genome of the plant [21–25]. Phenotypic and genetic changes following tissue culture also result from DNA methylation alterations, generally elevated mutation rates, and bursts of transposon activity [9, 26–31]. These genomic, genetic, and epigenetic changes are heritable in future generations, presenting a potential problem for subsequent studies as phenotypes caused by a transgene can be confounded with phenotypes resulting from the tissue culture process itself [28, 32–34].

Importantly the drivers of unintended but heritable changes following tissue culture are not uniform across species. For instance, although transposon bursts have been widely documented in many plant species emerging from tissue culture, this phenomenon was not detected in *Arabidopsis thaliana* plants [35]. In contrast, in maize (*Zea mays*), tobacco (*Nicotiana tabacum*), and rice (*Oryza sativa*), bursts of numerous transposon families have been observed following tissue culture [30, 36, 37]. Passage through tissue culture is also frequently associated with elevated mutation rate as well as changes in gene expression and genome structure [28, 38–40]. Stable transformation of solanaceous plants, such as the horticulturally important species tomato (*Solanum lycopersicum*), potato (*S. tuberosum*), bell pepper (*Capsicum annuum*), petunia (*Petunia spp.*), tobacco (*Nicotiana spp.*), and *Datura stramonium* requires tissue culture, despite unreproducible claims of other transformation methods [41]. However, the impact of tissue culture on genome structure, gene expression, and mutation rate in these species has not been characterized. This makes characterizing the genomic impacts of tissue culture on these plants important in order to contextualize subsequent genetic and genomic studies in these species.

Previously, we published a tissue-culture based transformation protocol for *D. stramonium* and demonstrated stable inheritance and expression of a green fluorescent protein (GFP) transgene [42]. To enable targeted engineering and breeding of *Datura stramonium*, and to examine the impacts of the passage through tissue culture on genomic structure, we sequenced, assembled, and characterized a reference genome of this species. We then resequenced the genomes of three third-generation (T3) transformant progeny of this plant and combined this with mRNA-seq of leaf tissue to determine the impact of tissue culture on the genome and on gene expression.

## Results

### *D. stramonium* has a moderately repetitive, average-sized genome for Solanaceae

Because individuals of *Datura* frequently vary in ploidy naturally, we assessed the ploidy of our reference-genome prior to assembly using Smudgeplot [43–47]. Raw sequencing reads supported this plant as having a diploid genome (Supplementary Fig. 1).

We produced an initial short-read assembly with ABySS and scaffolded, gap-filled, and polished this assembly with high-coverage, short reads and low coverage long reads (Table 1, Supplementary Results). After removing small contigs (≤500 bp), our assembly was 2.1Gbp and contained approximately 24% gaps. This resulted in a BUSCO score for the final assembly of 94.7%. The contig and scaffold N50 values are 13kbp and 164kbp, respectively. The largest contig and scaffold are 235kbp and 1.48Mbp, respectively (Table 1).

**Table 1:**
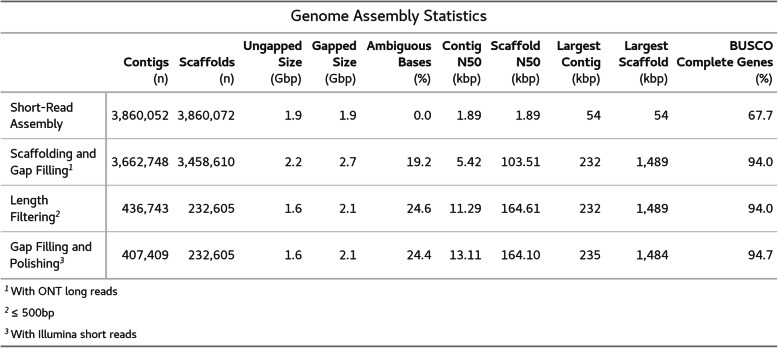
Genome Assembly Statistics. Summary statistics for the reference genome of *Datura stramonium*. Final version of the genome is shown on the last line. Contig and scaffold are shown as a count. Ungapped and Gapped sizes represent the total length in gigabasepairs of the assembled genome without or with ambiguous bases (Ns), respectively, introduced during scaffolding. Ambiguous bases are shown as a percentage of the total gapped genome size. Contig and scaffold N50 are shown in kilobase pairs as are the largest contig and scaffold

Following a preliminary repeat masking with RepeatModeler and RepeatMasker, we applied the Extensive de novo TE Annotator (EDTA) pipeline to achieve a more comprehensive and detailed inventory of transposable elements across this genome [48–50]. This pipeline annotated approximately 60% of the genome as transposable elements or repeats. A summary of repetitive elements delineated by superfamilies as defined by Wicker et al. is presented in Table 2 [51]. Over half of the annotated repetitive elements belong to the *Gypsy* superfamily of Long Terminal Repeat (LTR) retrotransposons, with unclassified LTRs and the *Mutator* superfamily of Terminal Inverted Repeat (TIR) DNA transposons making up the next two most numerous classes of repetitive elements. *Gypsy*-type LTRs also make up roughly a third of the genomes of several sequenced *Solanum* species, and the repetitive content of the genomes of *Capsicum annuum* and *C. chinense* are also approximately half *Gypsy*-type LTRs [52–55]. In relation to other sequenced Solanaceae genomes, this estimate of repetitive content for the assembled genome is comparable to that of *Nicotiana benthamiana* (61%) and *Petunia spp.* (60–65%), but much less than *Capsicum annuum* (76%), *S. lycopersicum* (72%), *N. tomentosiformis*, and *N. sylvestris* (75 and 72%, respectively) [55–59].

**Table 2:**
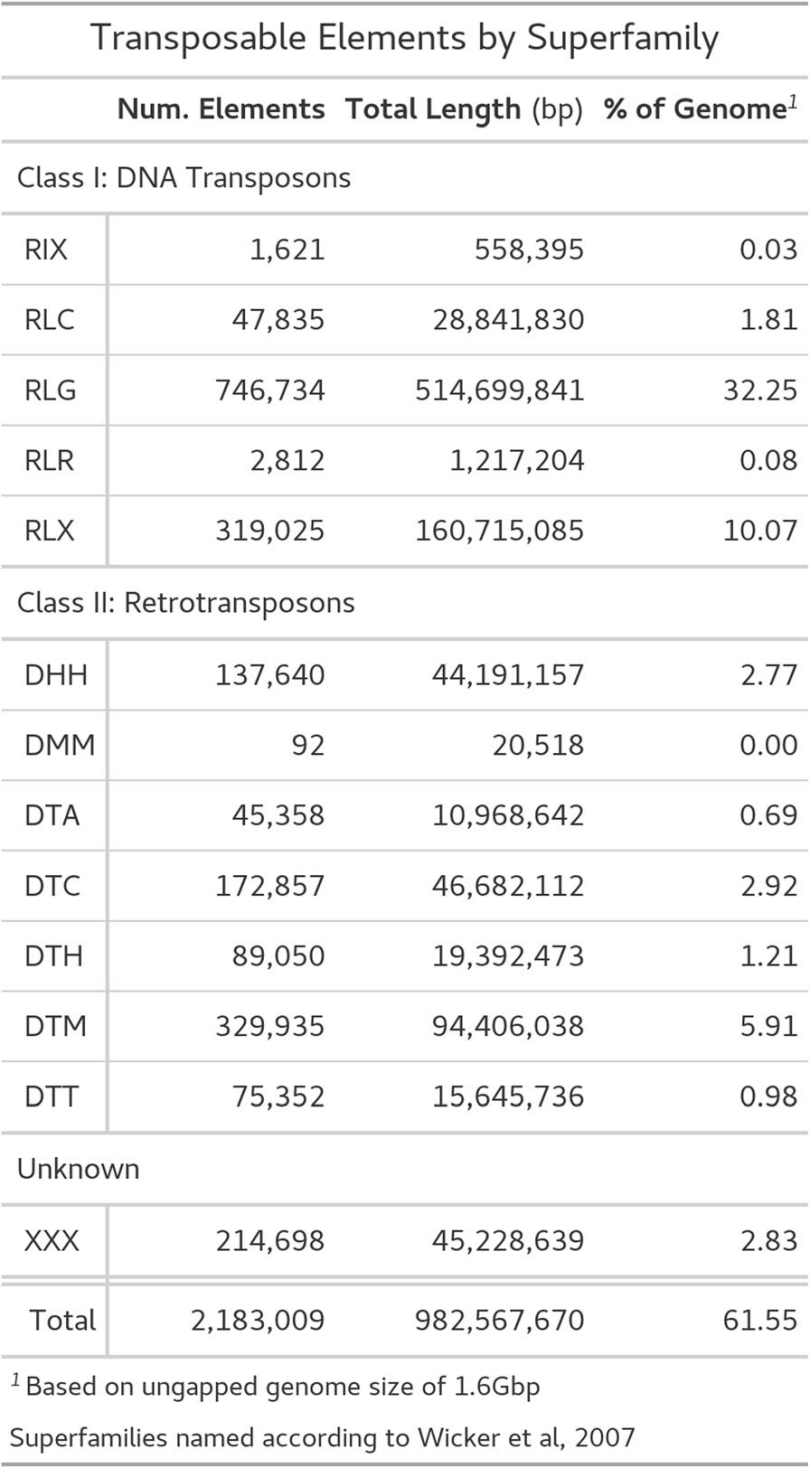
Transposable elements are broken down first by class then by superfamily (abbreviated according to Wicker et al, 2007)

**Fig. 1:**
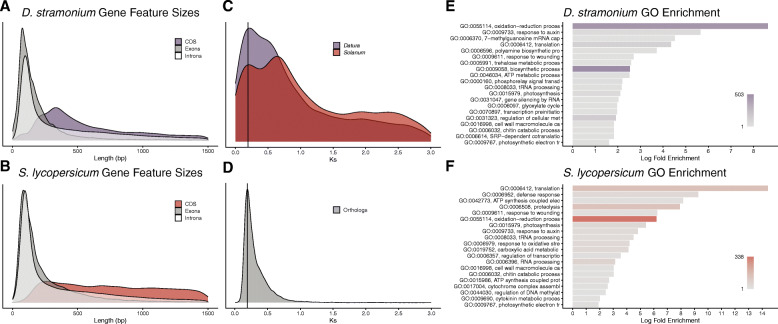
Summary of gene annotations. Density plots (**a**-**b**) of the sizes for total coding sequence lengths, individual exon lengths, and individual intron lengths for *D. stramonium* (**a**) and *S. lycopersicum* (**b**). Ks plots (**c**-**d**) showing the smoothed density of Ks values for paralogous genes (**c**) within *D. stramonium* (purple) or *S. lycopersicum* (red) and orthologous genes (**d**) between *D. stramonium* and *S. lycopersicum*. GO term enrichments for genes duplicated at the terminal branch of the phylogeny in Figure 3A for *D. stramonium* (**e**) and *S. lycopersicum* (**f**). GO term names have been truncated to fit available space, and bar colors correspond to the number of genes assigned to the given GO term, with a color scale shown in the lower right of each plot

Our nuclear genome annotation suggested 52,149 potentially protein-coding genes and an additional 1392 tRNA loci. This estimate of gene number is based on multiple sources of evidence including mRNA-seq transcript alignments, protein sequence alignments, and several *ab initio* gene prediction software packages. Despite this support, the total number of gene models is higher than closely related species such as tomato (34,075) and pepper (34,899) (Table 3) [52, 55]. Most of the identified genes have few exons, with a median exon number of 2 (mean 3.8), but a midasin protein homolog with 66 exons was annotated as well [60]. Across the genome, the median size of exons was 131 bp (mean 208 bp), while introns tended to be much larger with a median size of 271 bp (mean 668 bp) and a range between 20 bp and over 14 kb (Fig. 1a). Intron and exon sizes from our annotation mirror the sizes in *S. lycopersicum* (Fig. 1b), however the median length of gene coding sequences is much lower in *D. stramonium* (531 bp vs. 1086 bp).

**Table 3:**
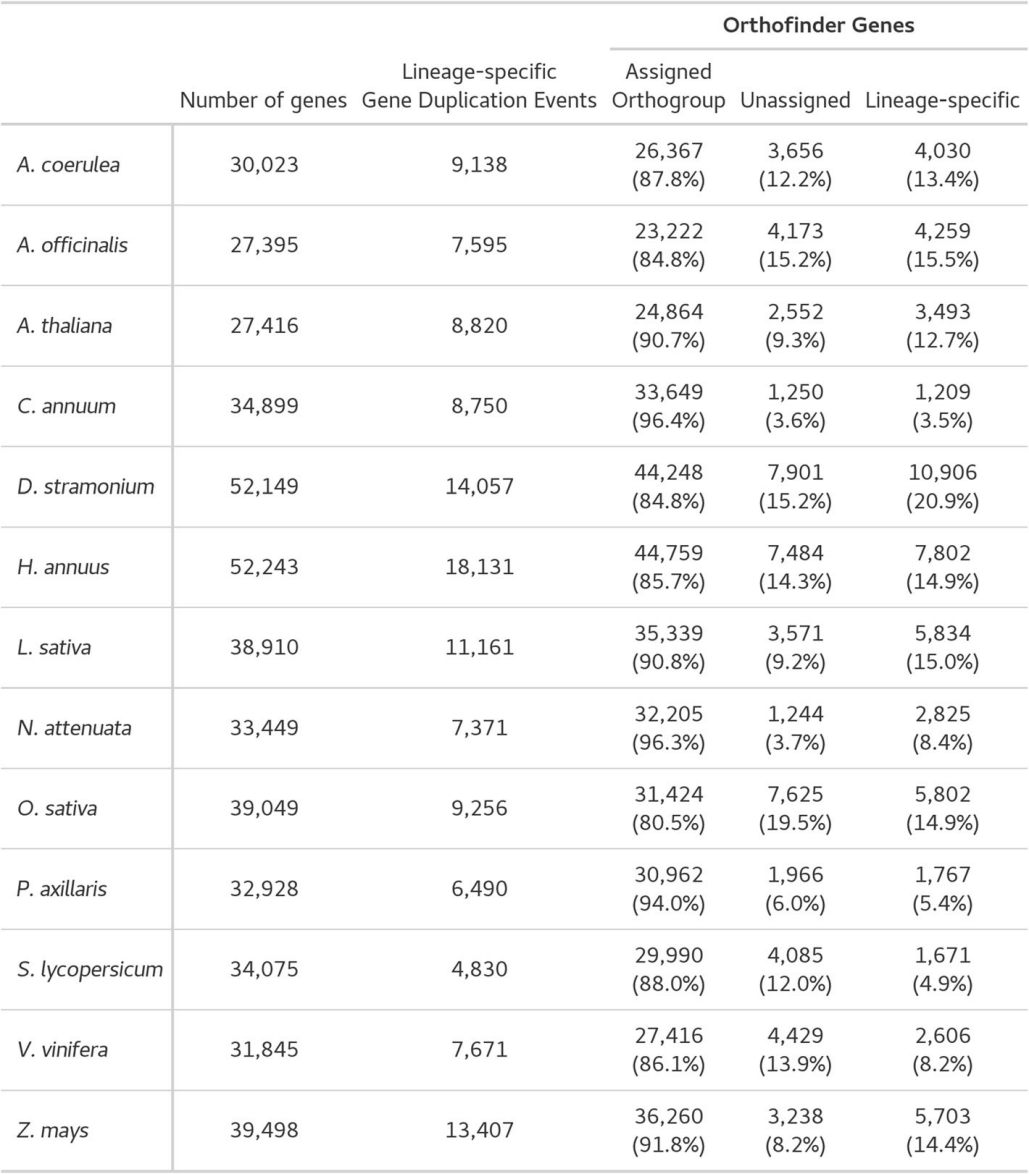
Orthofinder2 summary of ortholog search of 13 angiosperm taxa. Number of protein-coding genes used in the analysis, number of gene duplication events in this taxon not present at higher taxonomic levels, number of genes successfully assigned to an orthogroup (percent), number of genes not assigned to an orthogroup (percent), number of genes assigned to a lineage-specific orthogroup

### Heteroplasmy of chloroplast genome

**Fig. 2:**
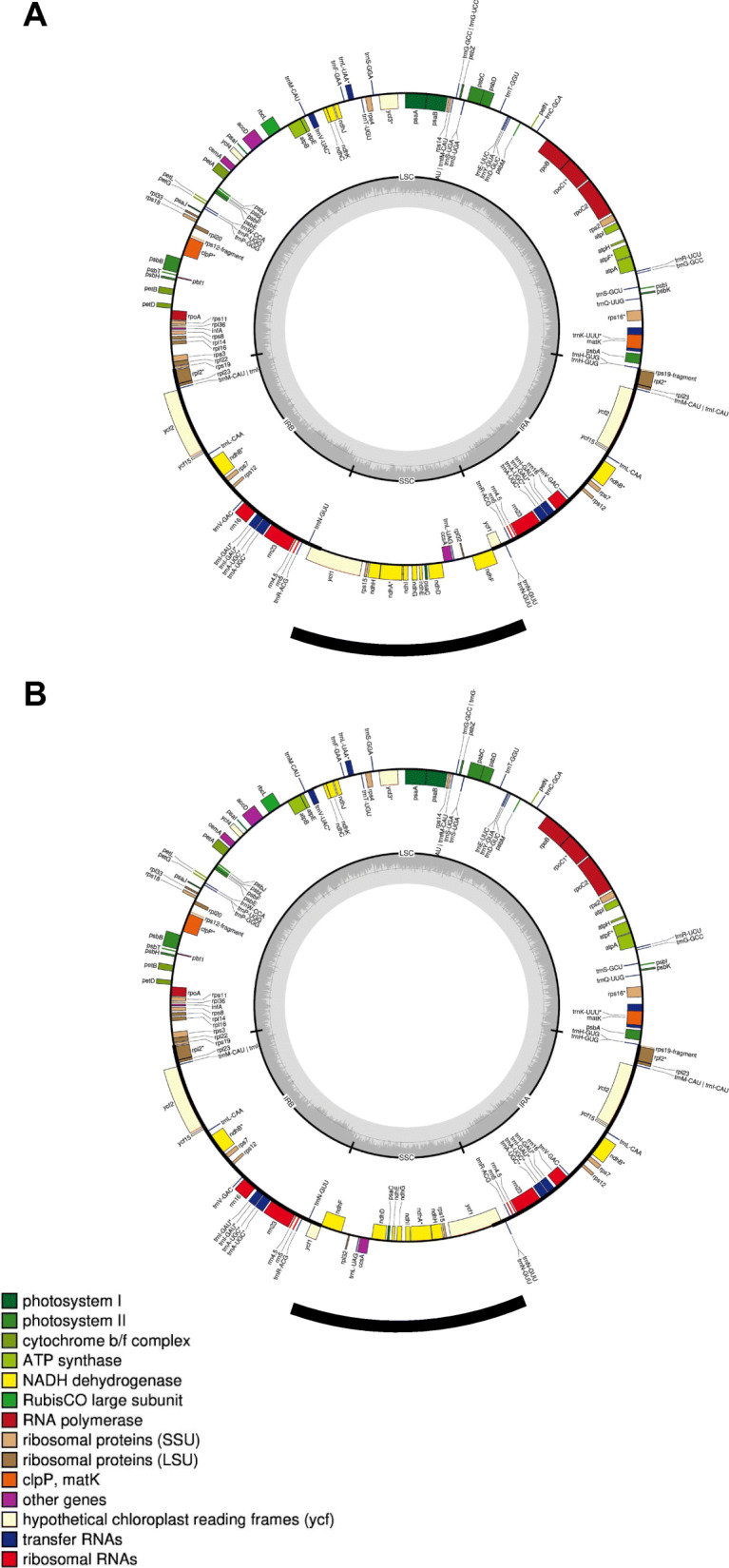
Assembled and annotated chloroplast genomes for *D. stramonium* showing the two inversion polymorphisms (**a** and **b**). The inverted small single-copy region is highlighted by the black sector below each circular genome. Annotated loci are plotted and labeled along the interior and exterior of the outermost circle. Loci are color coded by function as described in the legend in the lower left corner. The small single-copy, large single-copy, and inverted-repeat regions are delineated in the interior grey circles. Adapted from GeSeq output

We recovered sufficient reads to reconstruct the complete chloroplast genomes from our reference plant. The program GetOrganelle produced two distinct chloroplast genome assemblies, both of 155,895 bp. This corresponds well to the 155,871 bp size of the first published chloroplast genome of *D. stramonium* and to the 155,884 bp size from a pair of more recently published *D. stramonium* chloroplast assemblies [61, 62]. Following annotation with GeSeq, we noticed that our two assemblies differed from one another only in the orientation of their small single-copy region, but otherwise displayed the typical quadripartite structure of most angiosperm plastid genomes (Fig. 2) [63]. Inversion polymorphism within an individual is quite common among plants and has been documented many times since its discovery nearly 40 years ago [64]. Independent pairwise alignments of the small single-copy region and of the large single-copy region with both flanking inverted-region regions from our two genomes show no further polymorphisms. Because the assemblies from the more recent study by De la Cruz et al. have not been released, we aligned the complete sequence of the original assembly from the earlier Yang et al. publication to our assembly and observed a 99.97% identity [61, 62].

### Lineage-specific duplications cannot explain high gene number

To explore the possibility of lineage-specific gene number increases in *D. stramonium* as an explanation for the high gene number, we undertook a number of analyses to ascertain if this represented bona fide gene family expansions, whole genome duplications, or if it was an artifact of our annotation methods. Our mRNA-seq data from leaf tissue provided support for 62.8% of annotated genes, leaving approximately 19,900 genes with only theoretical evidence.

**Fig. 3:**
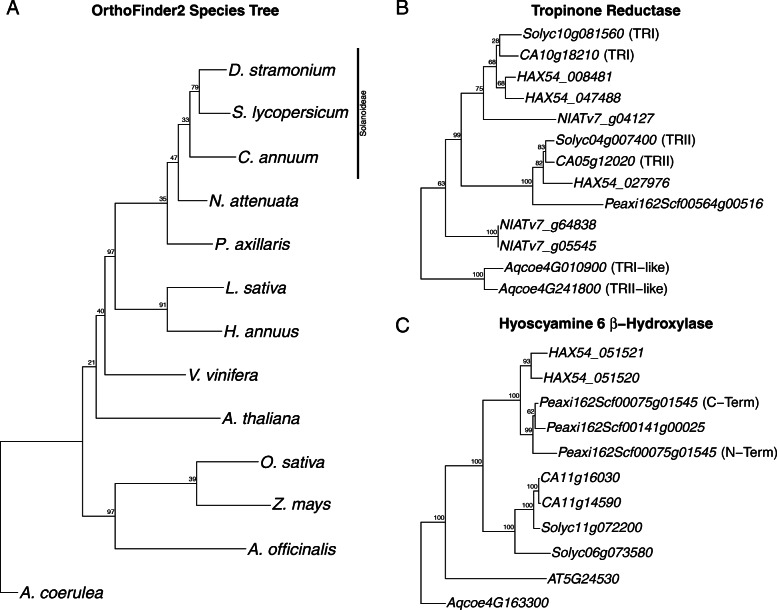
Phylogenetic trees representing (**a**) the species relationships inferred by OrthoFinder2, with the erroneously arranged Solanoideae clade highlighted by the black bar. The gene tree of putative tropinone reductase protein sequences (**b**), with previously published functional annotations of proteins in parentheses. The gene tree of putative hyoscamine 6 β-hydroxylase protein sequences (**c**) with the N-terminal and C-terminal domains of the petunia fusion protein annotated in parentheses

We used OrthoFinder2 to cluster protein sequences from *D. stramonium* and 12 other angiosperm species with sequenced genomes into orthologous groups and to identify gene duplication events [65]. The majority of these protein sequences were successfully grouped, and the inferred species tree from this analysis largely matched the previously established phylogeny of these angiosperm species (Fig. 3) [66–68]. Using all predicted proteins from the genome annotations, we found that approximately 12% of these proteins were present only in a single species, whereas only 482 proteins were present in a single copy across all 13 species. When examining duplication events mapped onto the species tree, *D. stramonium* stands out among Solanaceae for having 14,057 lineage-specific duplication events. This is much higher than the range among other solanaceous species, 4830 (*S. lycopersicum*) to 8750 (*C. annuum*) (Table 3). Across the entire species tree, *Helianthus annuus* has more lineage-specific duplications, with 18,131; however, this species has evidence of polyploidy events after its divergence from Solanaceae [69, 70]. The expansion events inferred in *D. stramonium* by OrthoFinder2 were not shared with the other members of Solanaceae, making them unlikely to have arisen during the hypothesized ancient Solanaceae triplication event [57, 71].

If the gene number expansion in *D. stramonium* represent a burst of recent lineage-specific expansions, then these paralogous genes should share higher sequence similarity with each other than with orthologous genes in other Solanaceae species. To examine this possibility and to estimate the relative age of gene number expansions, we plotted the frequency of synonymous substitutions (Ks) between all pairs of genes within both *D. stramonium* and *S. lycopersicum* as well as between all pairs of single-copy orthologs between these two species (Fig. 1c-d). Within both species, the leftmost peak in Ks values is around 0.19 (Fig. 1c), and this peak also corresponds to the peak in Ks values among single copy orthologs between the two species (Fig. 1d). We did not detect well-supported Ks peaks for paralogous genes in either species with lower Ks values than this, suggesting that neither *D. stramonium* nor *S. lycopersicum* have undergone detectable bursts of gene duplication since their divergence from one another. Taken together, the large number of genes without mRNA-seq support, without obvious orthologs in 12 other angiosperms, and without evidence of evolutionarily recent lineage-specific expansions suggest that the higher number of genes in *D. stramonium* compared to other Solanaceae is likely due to overestimates of gene number rather than a bona fide increase in gene number.

We performed a GO term enrichment analysis on all of the genes from lineage-specific duplications in *D. stramonium* and *S. lycopersicum* to look for trends among these genes (Fig. 1e-f). Between these species, many of the GO terms were very broad. For example, translation, oxidation-reduction processes, and response to auxin were enriched in both species’ datasets. Other categories of lineage-specific duplications were related to defense such as gene silencing by RNA, chitin catabolic processes, and response to wounding.

### Lineage-specific duplications of alkaloid biosynthesis genes

Because of the medicinal and pharmaceutical importance of *D. stramonium* tropane alkaloids, we examined our genome assembly and annotation for evidence of changes in copy number of tropane alkaloid biosynthesis genes. The tropane alkaloid biosynthesis pathway is fairly well characterized and most of the enzymes responsible for the creation of the predominant tropane alkaloids of *Datura spp.* have already been elucidated [72].

In the lineage-specific duplication events for *D. stramonium*, we detected significant enrichment for the polyamine biosynthetic processes GO term (Fig. 1e, GO:0006596, *p* = 1.9 × 10^− 4^). Polyamines, such as putrescine, are precursor molecules for the production of tropane alkaloids [72, 73]. The gene trees inferred by OrthoFinder2 also showed lineage-specific duplications in *D. stramonium* of the genes encoding the enzyme tropinone reductase I (TRI) (Fig. 3b). Tropinone reductases function on tropinone to shunt the biosynthetic pathway toward pseudotropine, and eventually, calystegines in the case of tropinone reductase II (TRII) or toward tropine and the eventual production of the pharmacologically important alkaloids atropine and scopolamine in the case of tropinone reductase I (TRI) [72]. These duplications were not observed in *S. lycopersicum* or *C. annuum*.

One further lineage-specific duplication appears to have occurred in *D. stramonium* for the biosynthetic enzyme hyoscyamine 6 β-hydroxylase (H6H, Fig. 3c). This enzyme converts hyoscyamine into a more potent and fast-acting hypnotic, scopolamine [74]. The two paralogous H6H loci in *D. stramonium* are arranged in a tandem array approximately 2 kb apart and share nearly 80% amino acid sequence identity. Our OrthoFinder search placed two *P. axillaris* genes in the same orthogroup as the *D. stramonium* H6H genes, but failed to find orthogroup members from any of the other 11 species. Other solanaceous genes identified via a BLAST search fall into a group separate from the petunia and *D. stramonium* genes, suggesting that these might not be true orthologs. Taken together, the duplications of two structural enzymes in the scopolamine biosynthetic pathway of *D. stramonium* confirm the importance of tropane alkaloid production in this *D. stramonium*.

### Impacts of tissue culture-based transformation

Previously we developed a tissue culture regeneration protocol for *D. stramonium* and used this to demonstrate the first stable transgenic transformants in the genus [42]. Because all transgenic transformation protocols for solanaceous plants developed thus far require a tissue culture phase, we sought to characterize the potential genomic and transcriptomic impacts of this process.

We resequenced the genomes of three plants derived from GFP-transformants in the 2019 study. All three individuals were derived from the same transgenic event and were propagated through single-seed descent of selfed plants for three generations after tissue culture. The estimated genome coverage for resequencing varied from 2-5x among the three plants. Overall, we detected over two million variants among the three transformants, with over half of the variants being SNPs. Indels ranged in size from 28 bp deletions to 22 bp insertions, but over 66% of indels were only ±1 bp. The vast majority of these polymorphisms were intergenic (74.3%, Table 4) with an additional 21.8% appearing proximally (±5 kb) upstream or downstream of coding regions. Only 1% of polymorphisms were present within exons and 2.8% were present in introns or at splice junctions. Of the exonic variants, about one third produced silent mutations while 64% created missense mutations. Nonsense mutations only accounted for 2.2% of variants.

**Table 4:**
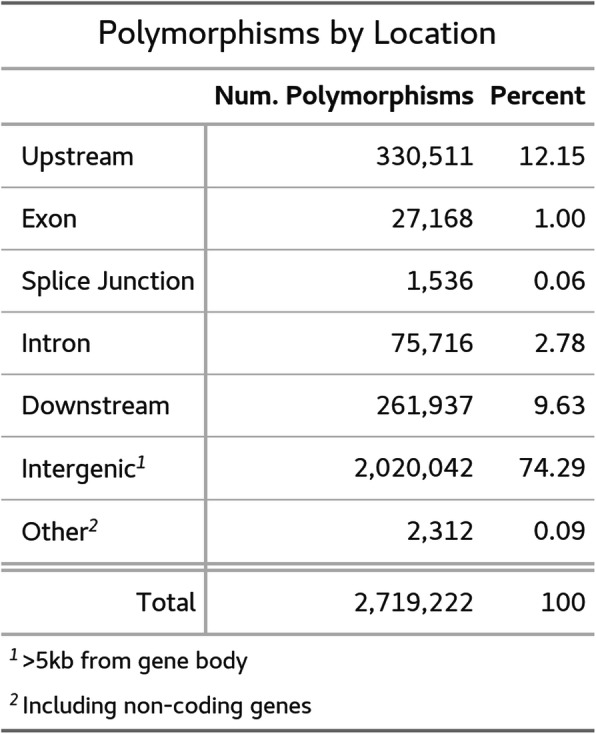
Total polymorphisms in the three resequenced GFP transformants classified by their location with respect to specific gene regions or intergenic regions

**Fig. 4:**
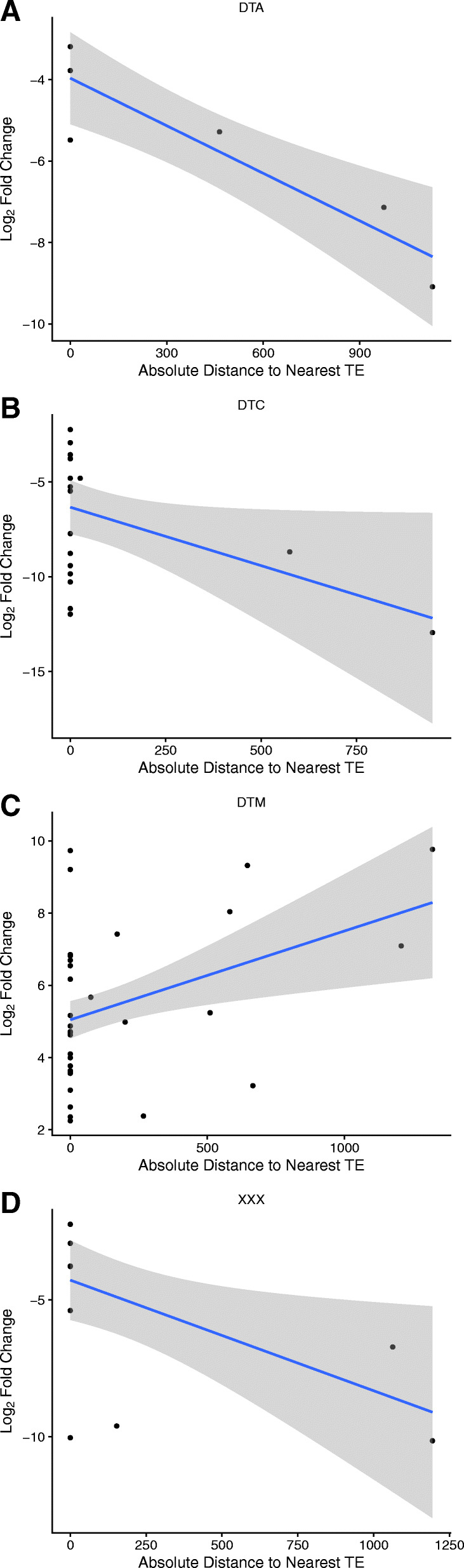
Linear relationship between absolute distance from the gene body to the nearest transposable element (in bp) and the log2 fold change of expression between the GFP transformants and wild-type plants. Downregulated differentially expressed genes for TIR/hAT (**a**), TIR/CACTA (**b**), and unknown (**d**) superfamilies, and upregulated differentially expressed genes for TIR/Mutator (**c**) superfamily elements

Although this analysis did not reveal strong evidence of duplicated genomic regions, we wanted to confirm that the transformants were still euploid diploids following tissue culture [75]. We used Smudgeplot to estimate the ploidy of each resequenced transformant from kmer frequencies [47]. Transformant #1 had the highest resequencing coverage and was determined to be a diploid regardless of the kmer length used. The two other resequenced transformants were assigned as diploids based on three of the four kmer lengths. Transformant #2 was determined to be a triploid with *k =* 15, while Transformant #3 was determined to be a triploid with *k =* 13. (Fig. 4).

Using all three transformants as replicates, we then conducted an mRNAseq experiment to look for potential differential expression of genes between the wild-type and transformed plants. With a FDR threshold of 0.01 and a log_2_ fold change threshold of 2, we were only able to detect 186 differentially expressed genes. Of these, 81 had lower expression in the GFP transformants compared to wild-type, and 105 had higher expression. We performed a GO term enrichment to determine if and to what extent the differentially expressed genes fell into distinct functional groups. The genes downregulated in the GFP transformants were slightly but significantly enriched for transport-related GO terms, specifically anion and organic acid transport (GO:0098656, GO:1903825, GO:1905039, GO:0006820, and GO:0009611). However only 1–2 genes fell into each of these partially overlapping categories. In contrast, the upregulated genes were generally enriched for regulatory GO terms, but spanned several regulatory terms from regulation of gene expression (GO:0010468) to regulation of nitrogenous compound metabolic processes (GO:0051171). These regulatory GO terms each represented between 8 and 10 genes.

We reasoned that changes in gene expression following tissue culture could be due to tissue-culture-induced mutations in regulatory regions or gene body regions important for transcript stability or transcription efficiency. We used the program snpEff to describe the impact of observed proximal tissue-culture induced polymorphisms might have on gene expression [76]. The program assigns polymorphisms into 26 categories describing their magnitude, effect, and location. Using a hypergeometric test, we asked if the differentially expressed gene set was enriched for any of the snpEff polymorphism categories compared to the rest of the genes in the genome. Four of the categories (impact_LOW, impact_MODERATE, effect_conservative_inframe_deletion, and effect_synonymous_variant) showed enrichment with *p*-values less than 0.05. For each of the four categories, we performed a linear regression, regressing the log_2_ fold change of expression on either the number of polymorphisms in each gene or simply the presence/absence of polymorphisms in each gene. In no case were any of these categories sufficient to explain changes in gene expression between the wild-type plants and GFP transformants (*p>* > 0.1).

In a separate attempt to explain the differentially expressed genes following tissue culture, we leveraged the transposable element inventory of the sequenced genome to look for correlations between differentially expressed genes and nearby transposable elements. Most genes contained both proximal (< 5 kb up- or downstream) as well as internal transposable elements. Here again, we performed a series of linear regressions, regressing log_2_ fold change of expression on distance to the nearest transposable element. We partitioned the data into several subsets for regression analyses. This included removing or including transposable elements located between the start and stop codons; only considering upstream transposable elements; using absolute distance from the gene body or signed distance from the gene body; and including all differentially expressed genes (DEGs), only upregulated DEGs, or only downregulated DEGs. All regressions were run both breaking the dataset apart by transposon superfamily and considering all transposable elements together. In all cases, distance to the nearest transposable element was capped at 5 kb. There were very few elements located further than this distance, and these rare data points had very high leverage in the regression analyses potentially inflating trends and *p*-values.

**Fig. 5:**
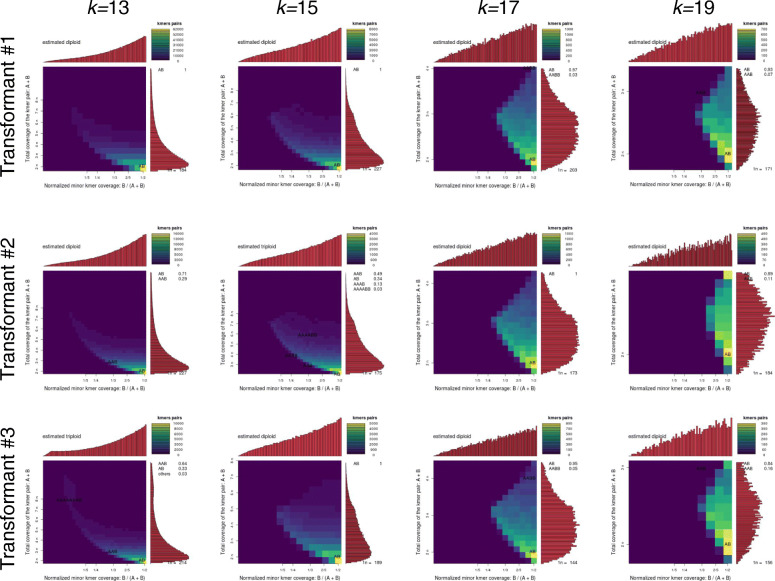
Smudgeplots using increasing k-mer lengths 13, 15, 17, and 17 (columns) on GFP transformants 1-3 (rows). Each smudegeplot is a 2D heat map showing the total coverage for a pair of k-mers differing by 1 bp versus the coverage of the minor k-mer in the pair as a fraction of the total coverage for the pair. Estimated ploidies are shown in the top left corner of each graph and the probability of various ploidies is shown on the right

When combining all transposable elements regardless of superfamily, we failed to see a statistically significant (*p* < 0.05) dependence between any of the distance metrics and log_2_ fold change of expression. We also saw no statistically significant dependence when examining helitrons (DHH) and all types of retrotransposons (RIX, RLC, RLG, and RLX). Additionally, looking only at upstream transposable elements likewise failed to show any statistical significance associations between log_2_ fold change of expression and distance. However, for several superfamilies of DNA transposons as well as for uncategorized transposable elements, we detected a statistically significant association between absolute distance to the nearest transposable element and log_2_ fold change. This association was present when partitioning the data into up- or downregulated DEGs such that, as distance to the nearest transposable element in the given superfamily increases, the magnitude of differential expression also increases (Fig. 5).

## Discussion

For well over a century, geneticists, biochemists, and evolutionary biologists have studied *Datura stramonium* for its interesting fruit and leaf phenotypes, startling alterations in ploidy, and useful production of various alkaloids [44, 73, 77, 78]. Here we continue this advance by providing a draft reference genome for the species. Characterizing this genome, we discovered lineage specific gene duplications for the first committed step in medicinally important tropane alkaloid synthesis and later in the final conversion step to produce the potent hypnotic scopolamine. We have used this new resource to determine the impacts on both mutation rate and gene expression from a recently developed transformation technique, which is critical for the further exploitation of the alkaloid biosynthetic pathway in *D. stramonium*.

Our reference genome assembly corresponds well to the previously estimated 2Gbp haploid genome size of *D. stramonium* based on flow cytometry and contains a very high percentage of BUSCO complete and single copy genes (Table 1) [79]. Combined with the associated draft annotation of protein coding genes, this resource will better enable future genetic and genomic studies in this species and perhaps allow us to revisit unanswered morphological and evolutionary questions from classical studies.

Our annotation suggests a higher than expected number of protein-coding genes in the genome of *D. stramonium* compared to the Solanaceae average of roughly 35,000 [55, 57, 58, 80, 81]. Multiple analyses suggest this is likely an overestimate. We considered lineage-specific gene expansion as an explanation, and compared protein sequences from *D. stramonium* and 12 other angiosperm species (Table 3). This analysis showed a high number of *D. stramonium* genes without apparent orthologs in the 12 other species. However, the percentage of genes present in only a single species (12%) along with the low number of single-copy universally present genes in this dataset (482) could suggest some inaccuracy associated with the orthology analysis, potentially caused by the large evolutionary distance between the 13 species and the polyploid history of several taxa.

Because of this uncertainty, we investigated the relative ages of the annotated genes in *D. stramonium* to investigate whether duplications specific to this lineage might explain the high gene count. An analysis of the pairwise rates of nonsynonymous substitutions (Ks) for all genes in both *D. stramonium* and *S. lycopersicum*, as well an analysis restricted to shared single-copy orthologs between these species, (Fig. 1c-d) likewise suggested that recent duplication does not underlie the high gene number. If the high number of genes present only in *D. stramonium* were due to a species-specific burst of gene duplication, this would be apparent as a peak with low Ks values, as these genes would all have very low numbers of synonymous substitutions when compared to their recent paralogs. Additionally, this species-specific peak would not be present in closely-related species and would have a lower average Ks value than orthologs between these species [82]. We did not observe such a peak in the Ks plots, therefore our data do not support the high number of lineage-specific genes in *Datura* resulting from bona fide lineage-specific gene duplication events.

Our mRNA-seq data and publicly available mRNA-seq data provide support for 35,470 genes. This leaves 16,679 predicted genes with no experimental verification. Although we expect that future studies of other tissues and conditions might provide evidence for some of these unsupported gene models, improved contiguity of the assembly in the future is likely to lead to a more reasonable estimate of gene number. Compared to the closely-related *S. lycopersicum*, the median length of individual *D. stramonium* coding sequences in our annotation is approximately half as long (531 bp vs 1086 bp), whereas the median length of exons was similar between the two species (Fig. 1e-f). This systematic difference in coding sequence length could be related to the fragmentary nature of our assembly, where a single gene could be split across multiple contigs and falsely treated as separate loci by gene prediction algorithms. A more complete assembly will also allow for more thorough repeat masking excluding ORFs from retroelements and other repetitive regions from the gene space. Indeed, a similar pattern was seen with the eggplant (*Solanum melongena*) genome, where the draft assembly estimate of 85,446 genes was revised downward significantly to 34,916 as later assemblies improved contiguity and mRNA-seq sampling [81, 83].

In terms of repetitive DNA content, our assembly suggests that *D. stramonium* is unremarkable amongst other Solanaceae with its 61% repetitive DNA, comparable to *Petunia* and *N. benthamiana*, and slightly lower than tomato, pepper, and several other tobacco species. Over half of the annotated repetitive elements belong to the *Gypsy* superfamily of Long Terminal Repeat (LTR) retrotransposons (Table 2). This results is in keeping with our knowledge of other closely related plants. Indeed *Gypsy*-type LTRs similarly make up about a third of the genomes of several sequenced *Solanum* species [53, 54, 71]. The repetitive portion of the *Capsicum annuum* and *C. chinense* genomes are also approximately half *Gypsy*-type LTRs; however, these genomes contain more repetitive DNA overall [52]. Within the Solanaceae family but outside the Solanoideae subfamily, which contains *Datura stramonium*, *Capsicum spp.* and *Solanum spp.*, *Gypsy* superfamily LTRs also make up much of the repetitive DNA. This superfamily alone comprises between one third and one half of the genomes of several *Nicotiana* species [80]. *Gypsy*-type LTRs are the most abundant superfamily of repetitive elements in the *Petunia axillaris* genome as well; however, *Copia*-type LTRs make up a nearly equal share of the genome, unlike in other solanaceous species [57].

These results should be interpreted with two caveats in mind. First, our assembly contains approximately 24% ambiguous bases, representing gaps of known size but unknown sequence between contigs. Precisely because our sequencing methods could not resolve these gaps, it is very likely that they correspond to highly repetitive regions of the genome such as centromeres, rDNA loci, or intergenic regions with nested/tandem transposable element insertions. Second, our scaffolds are not yet assigned to chromosome-scale linkage groups or pseudomolecules. Our current assembly comprises over 200,000 scaffolds; however, based on previous karyotyping studies and the assumed conservation of base chromosome number in the subfamily Solanoideae, we expect *D. stramonium* to have 12 haploid chromosomes (x = 12) [44, 68]. In the future, resolving these gaps with additional long-read sequencing, optical mapping, proximity ligation sequencing, or other techniques could achieve a more contiguous, chromosome-scale assembly. Such an assembly would also provide better evidence for genome size, repetitive DNA content, transposable element annotation, and gene annotation. The resolution of full-scale chromosomes would also enable a more precise characterization of structural variation following tissue culture. Overall however, our kmer-based Smudgeplot analysis, BUSCO duplicate genes score, and paralog Ks plots are all consistent with our reference genome deriving from a typical eudiploid plant and support its use in future genetic and genomic studies.

One of our key findings was the lineage-specific duplications in two tropane alkaloid biosynthetic genes. Early in this pathway, the enzyme tropinone reductase I (TRI) acts to shunt the production toward tropine and the derivative tropane alkaloids by competing with tropinone reductase II (TRII), which produces pseudotropine leading eventually to calystegine alkaloids [72, 84]. We show evidence for a lineage-specific duplication of TRI in *D. stramonium* that is not shared with the other members of the Solanoideae subfamily of Solanaceae that we examined (Fig. 3b). Following the formation of tropine, several other biochemical reactions can eventually lead to the production of hyoscyamine, a pharmaceutically important tropane alkaloid in its own right. However, many *Datura spp.* are known to accumulate the hyoscamine derivative, scopolamine, as the primary tropane alkaloid instead [85]. At this step in the biosynthesis pathway, we discovered the second lineage-specific gene duplication in *D. stramonium*, with a tandem duplication of hyoscyamine 6β-hydroxylase (*H6H*, Fig. 3c). This gene was successfully targeted in a previous effort to increase tropane alkaloid content in *Atropa belladonna* [5]*.* Our initial search for orthologs of these genes with OrthoFinder2 found two genes in *P. axillaris* and none in the other 11 species included in this study. Unlike the tandem duplicates in *D. stramonium*, the two petunia genes are located on different scaffolds. Remarkably however, one of the petunia genes (*Peaxi162Scf00075g01545)* appears to encode a fusion protein of two tandemly-arrayed *H6H* genes transcribed in-frame, which we split apart for our phylogenetic analysis to examine their evolutionary relationships independently. Interestingly, the two regions of the fused petunia genes do not appear most closely related to each other in our phylogenetic analysis, as could be expected if they were the result of a recent tandem duplication followed by a fusion. Instead, the C-terminal region of the fused complex appears quite similar to the unfused gene, suggesting that perhaps the evolutionary history of these *H6H* genes in Solanaceae is more complex. Our BLAST search did recover similar proteins among other solanaceous species, but these grouped distinctly in our phylogenetic analysis. This arrangement could be an artifact of narrow taxonomic sampling or possibly independent derivations from an ancestral protein of unknown function. Importantly, our dataset does not include other solanaceous species with notable production of tropane alkaloids, as we were unable to find assembled genomes for any of these species. Including sequences from genera such as *Atropa*, *Scopolia*, or *Hyoscyamus* could shed more light on the evolution of this enzyme and clarify this unlikely grouping of *Petunia* and *Datura* protein sequences. Broader sampling is likely to clarify the history of gene duplication and loss that could have led to the phylogenetic arrangement we observed.

Our previously published protocol for transformation of *D. stramonium* enabled more thorough functional genetic studies, but also carried with it the possibility of genomic changes induced by tissue-culture itself [42]. To better characterize these potential changes, we resequenced the genomes of three plants descended from a transformant in the original study. We detected several million polymorphisms (SNPs and indels) among the resequenced plants compared to the reference genome. This amounts to 1.16 × 10^− 3^ mutations per site, which is much higher than the estimated mutation rates following tissue culture in either *A. thaliana* (between 4.2 × 10^− 7^ and 24.2 × 10^− 7^ mutations per site) or *O. sativa* (5.0 × 10^− 5^ mutations per site) [38, 39]. Our analysis pipeline took PCR duplicates from library preparation and potential sequencing errors into account, so we expect that our analysis is detecting bona fide polymorphisms between the transformants and the reference genome. Our plants were allowed to self-pollinate prior to reference genome sequence and between tissue culture and genome resequencing, however our methods cannot rule out that some of these polymorphisms are due to standing heterozygosity in the resequenced plants not captured by the reference genome. We expect the mutation rate following tissue culture to be higher than normal, but we also know that mutation rates are not uniform across species or even cultivar boundaries [38, 39, 86]. We currently do not know the background mutation rate in untransformed *D. stramonium*, therefore our estimate could be further refined with that information and with long-term mutation-accumulation studies [87].

Although our transformants accumulated a large number of mutations compared to the reference genome, their impact appears low. The mutations following tissue culture were overwhelmingly found in intergenic regions of the genome. Only 27,000 exonic mutations are present across over two million mutations in the three individuals. However, nearly two thirds of these exonic mutations are not silent and could potentially affect protein function, secondary structure, etc. An analysis of changes to the epigenome, which is frequently connected with aberrant phenotypes of transformants, would likely be informative [7, 17, 88]. It is also possible that mobilization of transposable elements is responsible for some alteration in the transformants [89, 90]. This movement along with other large scale structural changes to the genome have been observed following tissue culture; however, we were unable to apply the computational tools to detect this, given the fragmentation of our assembly [91–93].

Despite these unknowns, it is encouraging that when we examined the transcriptomic impacts of tissue culture on our transformants, the results were negligible. Using our thresholds of differential expression (FDR < 0.01 and log_2_ fold change> 2), we were only able to call 186 genes as differentially expressed between the transformed and untransformed plants. We did detect significant GO term enrichment for certain classes of genes among the 186 differentially expressed genes, including regulatory terms and transmembrane transport. Our attempts to explain this small number of differentially expressed genes through correlation with polymorphisms or transposons did not produce robust results, though some weak association between magnitude of differential expression and distance to certain DNA transposons superfamilies was present and has been remarked on by other studies as well [94, 95]. High variation in gene expression levels among individuals could also contribute to the low number of statistically significant differentially expressed genes; however, this is unlikely to be a result of tissue culture here as all transformants in this study were derived from a single transformation event. Overall it seems that other factors not captured by our study could be behind the differential expression of this subset of genes.

## Conclusions

Our assembled and annotated 2 gigabasepair draft genome of *Datura stramonium* is the first in the genus and will be a valuable resource for others working on functional genomic studies in this system. Future work involving long-read sequencing technologies should improve the contiguity and annotation of this draft. Using this new resource along with mRNAseq and genome resequencing, we show that following tissue culture, mutation rates of transformed plants are quite high, but do not have a substantial impact on gene expression.

## Methods

### Plant material

#### Growth conditions

For genome sequencing, wild-type *Datura stramonium* seeds were obtained in 2013 from J. L. Hudson Seedsman (La Honda, California, USA), sown directly on soil, and grown under greenhouse conditions at the University of California, Riverside for three generations with self pollination to increase homozygosity prior to genome sequencing.

For genome resequencing and gene expression analyses of transgenic plants, we used GFP-transgene harboring seeds previously described in Rajewski et al. [42]. These seeds correspond to the second generation seed from individual T_1_–4, making these seeds three generations removed from tissue culture. We selected progeny of T_1_–4 based on its brighter GFP fluorescence than that of its siblings in order to aid screening. To increase germination efficiency, we dissected away the outer seed coat of these seeds. All plants for gene expression analyses and genome resequencing were maintained at 22 °C for 24 h under 100 μmol m^− 2^ s^− 1^ light conditions at a 16 h light and 8 h dark photoperiod.

For a wild-type gene expression analysis, we selected sibling seed of the genome sequenced individual, dissected the seed coat away, and germinated them under the same conditions as the GFP-transgenic seeds.

#### Nucleic acid isolation

For short read sequencing, we isolated DNA from a single developing leaf of one wild-type, greenhouse-grown *Datura stramonium* plant described above using the E.Z.N.A Plant DNA Kit (Omega Bio-tek, Norcross, GA) according to the manufacturer’s instructions, and quantified its purity and concentration using a biospectrometer (Eppendorf AG, Hamburg, Germany). In order to isolate high molecular weight DNA for Oxford Nanopore sequencing, we used a CTAB DNA extraction with several modifications to reduce shearing of genomic DNA [96, 97]. The DNA was stored at − 70 until needed for library construction.

For gene expression analyses, we collected one immature leaf (~ 3 cm in length) each from three wild-type and three plants harboring the GFP transgene. We snap froze this tissue in liquid nitrogen, ground each sample using steel BBs in a Retsch MM400 mixer mill (Haan, Germany), and isolated RNA with the RNeasy Plant Mini Kit (QIAGEN, Hilden, Germany). RNA isolation proceeded according to the manufacturer’s protocol except that the lysis step of this protocol was modified to use buffer RLC instead of RLT and supplemented with 2.5% (w/v) polyvinylpyrrolidone (PVP). We removed DNA contamination with an on-column RNAse-Free DNAse kit (QIAGEN, Hilden, Germany) according to the manufacturer’s protocol. The UCR Genomics Core assessed the integrity of the isolated RNA using an Agilent 2100 Bioanalyzer. We stored the material at − 70 °C.

### Reference genome sequencing

We used the SeqOnce Rapid DNA-seq preparation Kit (Beta Version 4.0d, SeqOnce Biosciences, Pasadena, CA) to prepare a DNA sequencing library. This library was sequenced across two partial Illumina NovaSeq 2x150bp runs at the University of California San Francisco Functional Genomics Core Facility, and produced 165Gbp of sequencing data, corresponding to ~100x haploid genome coverage. For long-read Oxford Nanopore sequencing, we used the high molecular weight DNA (greater than 28 kb) and the Ligation Sequencing Kit SQK-LSK109 (Oxford Nanopore, UK) to create a 1D sequencing library. We sequenced this on a MinION flow cell R9.4 to generate approximately 13Gbp of data (~9x haploid genome coverage). Read sizes ranged from 330 kb to 500b with a mean of 9.4 kb.

### Reference genome assembly and annotation

All scripts used to assemble and annotate this reference genome are available in a public Github repository (https://github.com/rajewski/Datura-Genome).

We first created several short-read only assemblies using ABySS (v2.0.2) with odd kmer sizes from 33 to 121 bp, but ultimately selected k = 101 as the optimal kmer size based on the assembly’s BUSCO score using the embryophyta version 9 lineage dataset [98, 99].

Following base calling by Guppy, we error-corrected the Nanopore reads using LoRDEC (v0.9) [100]. We then used the optimal ABySS assembly for several iterations of scaffolding, gap-filling, and polishing using LINKS (v1.8.4), RAILS (v1.5.1), and ntEdit (v1.3.0), respectively [101–103]. For LINKS scaffolding, we selected a relatively high kmer size of 19 bp because we were using error-corrected Nanopore reads. We scaffolded with insert sizes of 750 bp, 1 kb, 5 kb, 10 kb, 15 kb, 20 kb, 30 kb, 40 kb, 60 kb, 70 kb, 80 kb, 90kp, and 100 kb. Gap filling with RAILS also used the error-corrected LoRDEC reads. Polishing with ntEdit was run several times after each scaffolding or gap-filling step until the number of edits stabilized. The kmer size for ntEdit was 50 bp.

Prior to gene annotation, we used RepeatModeler (v1.0.11) and RepeatMasker (v4–0-7) to generate and soft mask a preliminary set of repetitive elements in the assembled genome [49, 50]. This set of repetitive elements was excluded from the subsequent gene annotation.

We applied the funannotate pipeline (v1.6.0) to annotate the assembled genome for protein coding genes and tRNAs [104]. Funannotate is a wrapper for several evidence-based and ab initio gene prediction softwares but also includes convenience scripts to simplify submission of genome annotations to data repositories such as NCBI. To train the gene predictors, we provided publicly available RNA sequencing data from NCBI SRA accession SRR9888534, along with the *D. stramonium* reads from medplantrnaseq.org, and mRNA-seq reads generated for the differential gene expression analyses (below). Following the training step, funannotate ran AUGUSTUS (v3.3), GeneMark-ETS (v4.38), SNAP, and GlimmerHMM (v3.0.4) [105–108]. Funannotate combined these gene prediction outputs with alignments of transcripts, generated by Trinity (v2.8.4) and PASA (v2.3.3), and protein evidence and passed them to EVidenceModeler (v1.1.1) which produced a well-supported annotation of protein coding genes [109–111]. Separately, tRNAscan-SE (v2.0.3) searched for and annotated tRNA loci in the assembled genome [112].

Once the annotation of protein coding genes and tRNA loci was completed, we used the Extensive de novo TE Annotator (EDTA) pipeline to create a more thorough annotation of TIR, LTR, and helitron transposable elements [48]. This analysis made use of the gene annotation information to remove potentially protein coding loci from the transposable element inventory.

We used GetOrganelle (v1.7.1) to assemble both organellar genomes [113]. For the plastid genome, we used the previously published *D. stramonium* plastid assembly (GenBank accession NC_018117) as an alignment seed [61]. To annotate genes as well as the large and small single copy regions and inverted repeat regions, we used GeSeq [63]. For the mitochondrial genome, we used the *S. lycopersicum* mitochondrial genome (Genbank accession NC_035963) as the seed. To determine the similarity to the reference plastid genome, we aligned with the full-length plastid genomes with MAFFT [114].

We deposited the raw sequencing reads used to assemble this genome in the SRA under NCBI Bioproject PRJNA612504. This Whole Genome Shotgun project has been deposited at DDBJ/ENA/GenBank under the accession JACEIK000000000.

Summaries of gene features and transposable elements proceeded with custom R scripts that are available in the public GitHub repository.

### Ortholog analyses

To determine orthologues among the 13 species, we used OrthoFinder2 [65]. This analysis included three members of the subfamily Solanoideae, *D. stramonium*, *Solanum lycopersicum*, and *Capsicum annuum*; two more distantly related members of Solanaceae, *Nicotiana attenuata* and *Petunia axillaris*; two non-solanaceous asterids, *Helianthus annuus* and *Lactuca sativa*; three rosids, *Vitis vinifera*, and *Arabidopsis thaliana*; two grasses, *Zea mays* and *Oryza sativa*, one non-grass monocot, *Asparagus officinalis*; and finally, the early-diverging angiosperm *Aquilegia coerulea* [55, 57, 58, 69, 80, 115–121]. For non-solanaceous species, we downloaded the reference proteomes and reference transcriptomes from Phytozome (v13). References for *D. stramonium* were generated in this study, those for *S. lycopersicum*, *C. annuum* and *P. axillaris* were downloaded from Sol Genomics Network (http://solgenomics.net/), and those for *N. attenuata* were downloaded from the *Nicotiana attenuata* Data Hub (http://nadh.ice.mpg.de/NaDH/).

For gene tree construction, we used either the loci from the OrthoFinder2 clustering, or, in the case of H6H, added additional loci based on BLAST searches with OrthoFinder2 output protein sequences as queries [122]. We then aligned these protein sequences with MAFFT (v7.471) and constructed phylogenetic trees with RAxML-NG (v0.9.0) using the JTT + Γ + I model and 1000 bootstraps [114, 123].

For Ks estimates between and within *D. stramonium* and *S. lycopersicum*, we used the wgd software suite’s tools for all-vs-all protein searches, MCL clustering, and Ks distribution calculation [124]. We included the options *--nostrictcds* and *--ignorestop* during the all-vs-all protein searches to avoid various formatting issues with the publically available transcriptome sequence files. In the Ks distribution calculations, we also passed a proteome sequence file instead of relying on automatically translated transcriptomes. We plotted output data from the Ks distributions using a custom R script available on our public GitHub repository. To obtain estimates of constituent Ks peaks within the Ks distributions we also used the *wgd mix* program’s Bayesian Gaussian mixture model function to decompose the distributions, determine peak Ks values, and Ks peak weights.

For lineage specific duplication events in *D. stramonium*, *S. lycopersicum*, and *A. thaliana*, we conducted GO enrichment analyses of duplicated genes using a custom R script. For consistency, this script used custom GO annotations for the proteome of each of the three species, which we generated using InterProScan (v5.45–80.0) [125].

We used custom R scripts with help from the phytools and ggtree packages to plot and annotate phylogenetic trees [126, 127].

### Genome Resequencing and polymorphism analysis

The UCR Genomics Core constructed DNA-sequencing libraries for genomic DNA from the three GFP transgene-containing plants using the NEBNext Ultra II FS DNA Library Prep Kit for Illumina and sequenced them to approximately 5x haploid genome coverage in a 2x75bp Illumina NextSeq run. The raw DNA-seq reads were deposited in the SRA under BioProject PRJNA648005.

We mapped the reads for each plant back to the reference genome using BWA MEM, then removed duplicates and flagged discordant or split reads with SAMBLASTER [128, 129]. We then used FreeBayes and LUMPY as implemented by SpeedSeq to call SNPs and structural variants, respectively, between the reference genome and the resequenced transformants [130–132]. Subsequently, we applied both snpEff and bcftools to summarize the variants detected [76].

### Gene expression analysis

We prepared mRNA-sequencing libraries from RNA of the GFP transgene-containing plants and three wild-type plants using the NEBNext Ultra II Directional RNA Library Prep Kit for Illumina, and sequenced these on the same 2x75bp Illumina NextSeq run as the DNA sequencing libraries. This produced approximately 33 million reads for each plant. The raw RNA-seq reads were deposited in the SRA under BioProject PRJNA648005.

The demultiplexed RNA-seq data were trimmed with TrimGalore. The trimmed reads were mapped to the assembled reference genome using STAR (v2.5.3a) in a single pass using splice junctions annotated from the reference genomes [133, 134]. We then passed these counts directly into DESeq2 to identify differentially expressed genes between the two genotypes of plants [135]. A list of these genes is provided in an additional file (Supplementary File 1) and the script used to perform the differential expression analysis is included in our public GitHub repository. We generated a subset of genes with proximal transposable elements using our transposable element annotation and bedtools intersect. A list of these genes and the distance to the nearest transposable element is provided in an additional file (Supplementary File 2). All correlational analyses between gene expression and polymorphisms or transposable elements, including linear regressions and hypergeometric tests were conducted, summarized, and plotted using custom R scripts available in our public GitHub repository.

## Supplementary Information

**Additional file 1: Supplementary Fig. 1**

**Additional file 2: Supplementary Results**

**Additional file 3: Supplementary File 1**

**Additional file 4: Supplementary File 2**

## Data Availability

The datasets generated during the current study are available under the NCBI Bioproject accessions PRJNA612504 and PRJNA648005. Additionally, *D. stramonium* RNA-seq files used for genome annotation are available under NCBI SRA accession SRR9888534 and at https://medplantrnaseq.org/. Scripts to analyze this data are available in a GitHub repository at https://github.com/rajewski/Datura-Genome.

## Abbreviations

bp: Basepair
BUSCO: Benchmarking University Single-Copy Orthologs
DEG: Differentially Expressed Gene
DHH: Helitron
DMM: *Maverick*-type DNA Transposon
DTA: *hAT*-type DNA Transposon
DTC: *CACTA*-type DNA Transposon
DTH: *PIF-Harbinger*-type DNA Transposon
DTM: *Mutator*-type DNA Transposon
DTT: *Tc1-Mariner*-type DNA Transposon
FDR: False Discovery Rate
GFP: Green Fluorescent Protein
GO: Gene Ontology
H6H: Hyoscyamine 6 β-hydroxylase
indel: Insertion-Deletion
Ks: Synonymous Substitution
LTR: Long Terminal Repeat
mRNA: Messenger RNA
rDNA: Ribosomal DNA
RIX: Unidentified LINE Retrotransposon
RLC: *Copia*-type LTR Retrotransposon
RLG: *Gypsy*-type LTR Retrotransposon
RLR: *Retrovirus*-type LTR Retrotransposon
RLX: Unidentified LTR Retrotransposon
SNP: Single Nucleotide Polymorphism
T-DNA: Transfer DNA
TE: Transposable Element
TIR: Terminal Inverted Repeat
TRI: Tropinone Reductase I
TRII: Tropinone Reductase II,
XXX: Unknown Transposon
