# Supplementary figures and images for: Datura genome reveals duplications of psychoactive alkaloid biosynthetic genes and high mutation rate following tissue culture
Source: BMC Genomics. 2021 Mar 22;22:201. doi: 10.1186/s12864-021-07489-2 (PMC7986286; doi:10.1186/s12864-021-07489-2)

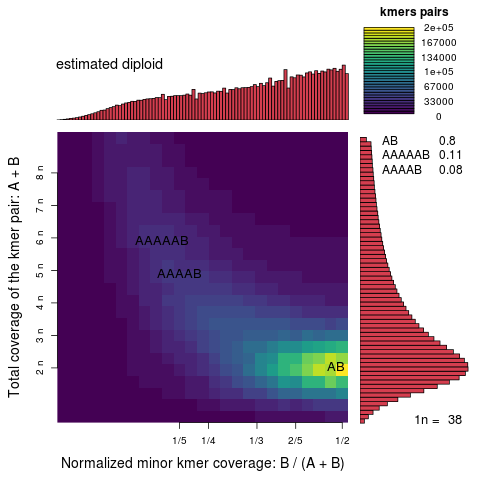

Supplement: Supplementary file 1 — Additional file 1: Supplementary Fig. 1 [file 12864_2021_7489_MOESM1_ESM.png]
