# Supplementary material for: Datura genome reveals duplications of psychoactive alkaloid biosynthetic genes and high mutation rate following tissue culture
Source: BMC Genomics. 2021 Mar 22;22:201. doi: 10.1186/s12864-021-07489-2 (PMC7986286; doi:10.1186/s12864-021-07489-2)
Supplement: Supplementary file 2 — Additional file 2: Supplementary Results [file 12864_2021_7489_MOESM2_ESM.pdf]

# Supplementary Results

## Genome Assembly

The short-read only assembly with ABySS produced a highly fragmented 1.9Gbp assembly with over three million contigs, 85% of which were shorter than 500bp (Table 1). This draft assembly size corresponded well to the haploid genome size estimate of 2.0Gbp determined by flow cytometry [49]. The completeness of this draft is also supported by the BUSCO value for complete, single-copy genes of 67.7% with an additional 14.2% fragmented genes. Due to the large number of very small contigs, the contig N50 was very low, 1.89kbp (Table 1). These values were the best among the various ABySS assemblies across all kmer values, and we proceeded with this assembly for further analysis.

We next used error-corrected, low-coverage long reads to scaffold this assembly and improve contiguity. In contrast to contigs, scaffolds routinely contain ambiguous bases (Ns) representing estimated gaps between contigs. Thirteen rounds of long-read scaffolding, long-read gap filling, and short-read polishing drastically improved most metrics of assembly quality. The largest scaffold increased from approximately 53kbp to nearly 1.5Mbp, while the largest contig increased to 232kbp. Similarly, the scaffold and contig N50 values increased from 1.89kbp to 103.5kbp and 5.4kbp, respectively. Because the long-read coverage was low (~7x), gap filling was only partially successful and the scaffolded assembly contained approximately 19% ambiguous bases, representing gaps between contigs in the scaffolds.

The extremely large number of small contigs (≤500bp) presented a number of computational and biological problems, in addition to negatively skewing assembly quality metrics. Working with a large number of small contigs drastically increased computing time for assembly, scaffolding, polishing, and annotation. Furthermore, their inclusion did not affect BUSCO scores, which remained at 94% after their removal, indicating that their contribution to

the genic portion of the genome is negligible. Finally, genome assembly submissions to many online repositories such as NCBI limit contig size to 500bp or larger. For these reasons, after long-read scaffolding, we filtered the assembly to include only contigs and scaffolds larger than 500bp.
